# Comparison of catheters or new arteriovenous fistulas for commencement of haemodialysis in pregnant women with chronic kidney disease: an international observational study

**DOI:** 10.1007/s40620-022-01288-y

**Published:** 2022-03-28

**Authors:** Shilpanjali Jesudason, Erandi Hewawasam, Brona Moloney, Rachel Tan, Joule Li, Hannah Blakey, Kate Bramham, Matthew Hall, Rajiv Juneja, Elizabeth Jarvis, Liz Lightstone, Graham Lipkin, Michelle A. Hladunewich

**Affiliations:** 1 Central Northern Adelaide Renal and Transplantation Services (CNARTS), Royal Adelaide Hospital, Port Road, Adelaide, Australia; 2 Faculty of Health and Medical Sciences, University of Adelaide, Adelaide, Australia; 3 Australia and New Zealand Dialysis and Transplant (ANZDATA) Registry, South Australian Health and Medical Research Institute (SAHMRI), Adelaide, Australia; 4 Renal Unit, Flinders Medical Centre, Adelaide, Australia; 5 Renal Medicine-University Hospitals Birmingham NHS Foundation Trust, Queen Elizabeth Hospital Birmingham, Birmingham, UK; 6 Department of Women and Children’s Health, School of Life Course Sciences, King’s College London, London, UK; 7 Renal and Transplant Unit, Nottingham University Hospitals, Nottingham, UK; 8 Wesley Medical Centre, Auchenflower, QLD Australia; 9 Centre for Inflammatory Disease, Department of Immunology and Inflammation, Faculty of Medicine, Imperial College London, London, UK; 10 Divisions of Obstetric Medicine and Nephrology, Department of Medicine Sunnybrook Health Sciences Centre, Temerty Faculty of Medicine, University of Toronto, Toronto, ON Canada

**Keywords:** Vascular access, Dialysis, Pregnancy, Arteriovenous fistula, Kidney disease

## Abstract

**Background:**

Evidence surrounding vascular access options for commencing dialysis in pregnancy complicated by chronic kidney disease (CKD) is limited. Creation of new arteriovenous fistulas (AVFs) in pregnant women is rare.

**Methods:**

Retrospective cohort study of approaches to vascular access in pregnancy in centres in Australia, the United Kingdom (UK) and Canada (2002–2018).

**Results:**

Twenty-three women with advanced CKD commenced dialysis in pregnancy (n = 20) or planned to commence (n = 3). Access at dialysis start was a tunnelled catheter (n = 13), temporary catheter (n = 1), AVF created pre-conception but used in pregnancy (n = 3) and AVF created during pregnancy (n = 3). No women commencing dialysis with an AVF required a catheter. No differences in perinatal outcomes were observed comparing AVFs and catheters at dialysis commencement. No AVFs were created in pregnancy in Canadian women. From Australia and the UK, 10 women had a new AVF created in pregnancy, at median gestation 14.5 weeks (IQR 12.5, 20.75). Four women still needed a catheter for dialysis initiation and 3 eventually used the new AVF. Six AVFs were successfully used in pregnancy at median gestation 24 weeks (IQR 22.5, 28.5), 2 were successfully created but not used and 2 had primary failure. No catheter-associated complications were identified except one episode of catheter-related sepsis.

**Conclusions:**

Catheter-related complications were minimal. In selected women, with sufficient pre-planning, an AVF can be created and successfully used during pregnancy to minimise catheter use if preferred. Pre-conception counselling in advanced CKD should include discussing vascular access options reflecting local expertise and patient preferences.

**Graphic abstract:**

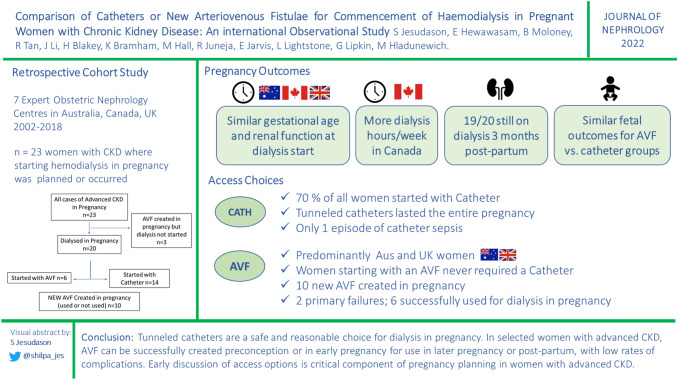

## Introduction

Pregnancy in women with advanced chronic kidney disease (CKD) is a rare but challenging scenario, requiring intensive management to optimise maternal and fetal outcomes. Women may require commencement of dialysis in pregnancy to facilitate lowering urea which has been associated with improved fetal outcomes [1, 2]. Rates of successful births in dialysed women have increased over time [3, 4] and outcomes have improved, particularly when treated with intensive haemodialysis [5]. There have been shifts in paradigms of clinician support and understanding of patient perspectives and desire for successful pregnancy [6–8]. Reliable, well-functioning vascular access is of paramount importance. However, little is known about practices and outcomes for vascular access choices for haemodialysis during pregnancy.

Vascular access choice for initiating haemodialysis in pregnancy will be informed by local practices or experiences of clinicians and patient factors including personal preferences [9]. Vascular catheters are unavoidable when urgent dialysis is required and preferred for chronic dialysis in non-pregnant patients in many centres [10]. Other centres have a “fistula first” policy in non-acute settings and lower rates of long-term catheter use. In women with advanced CKD, creating an arteriovenous fistula (AVF) before pregnancy or early in pregnancy can facilitate catheter avoidance, but requires sufficient pre-planning to allow time for maturation. Concerns regarding aneurysms, thrombosis and haemodynamic shunting in the context of massive physiological changes in pregnancy may discourage AVF creation. Alternately, concerns regarding catheter-related infection, radiation exposure to confirm position, catheter thrombosis and lower flow rates than AVF may lead to avoidance of planned vascular catheters in pregnancy [11, 12].

Data regarding outcomes of these two different approaches to vascular access for potential dialysis commencement in pregnant women with CKD are limited, and current guidelines do not contain specific recommendations [13]. Whether to create an AVF remains an important clinical question in complex, high-risk pregnancies where dialysis may eventually be required. However, creating or using a new AVF during pregnancy has been rarely reported [8]. This study describes experiences and outcomes for vascular access types for new haemodialysis initiation in pregnancy from centres in Australia, Canada and the United Kingdom (UK), in women with advanced CKD.

## Materials and methods

### Study population

This was a retrospective case series with data obtained from multiple obstetric nephrology services from 2002 to 2017. Australia: Royal Adelaide Hospital, Flinders Medical Centre, Women and Children’s Hospital, Adelaide. United Kingdom: King’s College Hospital, London; Nottingham University Hospitals, Nottingham; Queen Elizabeth Hospital, Birmingham; Canada: Sunnybrook Health Sciences Centre.

We included women who had a new AVF created during pregnancy; women who had a pre-existing AVF used for the first time during pregnancy; and women who had a vascular catheter inserted for new initiation of haemodialysis during pregnancy. We excluded women established on haemodialysis prior to pregnancy or with acute kidney injury in pregnancy.

### Data collection

Cases were identified by site investigators via existing pregnancy datasets where available and/or clinician recall by investigators and other clinicians at each centre. Data were extracted by local investigators from all available electronic and paper medical and laboratory records. Demographic, laboratory, clinical and outcomes data were collected into a standardised database. SJ, EH and BM collated de-identified data from centres for analysis.

### Statistical analysis and reporting

Categorical data were presented as frequencies and percentages. Groups were compared using Fisher’s Exact test. Where case numbers were insufficient for meaningful comparative analysis, only descriptive data are presented. Continuous data were presented either as mean ± standard deviations (SD) or median and interquartile range (IQR) as appropriate. Groups were compared using either one-way analysis of variance (ANOVA) or Kruskal–Wallis H test, as appropriate. Data were analysed using Stata SE (version 16.0) software. Values were considered statistically significant where p < 0.05. The study was reported in accordance with the Strengthening the Reporting of Observational Studies in Epidemiology Statement https://www.strobe-statement.org/.

## Results

### Haemodialysis initiation during pregnancy in women with CKD

#### Maternal characteristics

We identified 23 women in whom the indication for considering haemodialysis was CKD, either presenting or progressing in pregnancy. Maternal characteristics are summarised in Table 1. Canadian mothers were significantly older at conception than mothers from Australia or the UK. Overall, the cohort had predominantly Caucasian (white) or Asian (South East Asian) ethnicity. Kidney failure was most commonly due to vesico-ureteric reflux and IgA nephropathy. Other causes included systemic lupus erythematosus (SLE), tuberculosis-related interstitial nephritis, ANCA-associated vasculitis, single kidney, polycystic kidney disease, scleroderma, focal segmental glomerulosclerosis, and mesangial-proliferative glomerulonephritis. One woman had an existing transplant with advanced graft dysfunction at conception. Fourteen women had pre-existing hypertension, three women had cardiovascular disease.

**Table 1:** Maternal demographic and clinical characteristics according to country

Characteristics	Australia n = 6	Canada n = 9	UK n = 8	All Cases N = 23	p value
Maternal age at conception, years, mean (SD)	30.0 (2.3)	33.9 (2.7)	24.8 (5.6)	29.7 (5.5)	< 0.001
Ethnicity, n (%)					–
Caucasian	2 (33.3)	1 (11.1)	5 (62.5)	8 (34.8)	
Asian	2 (33.3)	4 (44.4)	3 (37.5)	9 (39.1)	
Indigenous	1 (16.7)	1 (11.1)	0 (0)	2 (8.7)	
African American	0 (0)	2 (22.2)	0 (0)	2 (8.7)	
Other	1 (16.7)	1 (11.1)	0 (0)	2 (8.7)	
Pre-existing hypertension, n (%)	6 (100)	5 (55.6)	3 (37.5)	14 (60.9)	0.06
Primary kidney disease, n (%)					–
SLE	2 (33.3)	1 (11.1)	0 (0)	3 (13.0)	
IgA nephropathy	1 (16.7)	4 (44.4)	0 (0)	5 (21.7)	
Vesico-ureteric Reflux	0 (0)	1 (11.1)	6 (75.0)	7 (30.4)	
Other	3 (50.0)	3 (33.3)	2 (25.0)	8 (34.8)	
Access at dialysis initiation in pregnancy, n (%)^a^					0.18
AVF	2 (33.3)	1 (11.1)	3 (60.0)	6 (30.0)	
Temporary catheter	1 (16.7)	0 (0)	0 (0)	1 (5.0)	
Tunnelled catheter	3 (50.0)	8 (88.9)	2 (40.0)	13 (65.0)	
AVF used at any time, n (%)^a^	5 (83.3)	1 (11.1)	3 (60.0)	9 (45.0)	–
Catheter used at any time, n (%)^a^	4 (66.7)	8 (88.9)	2 (40.0)	14 (70.0)	0.18
Serum creatinine at initiation dialysis (µmol/l), median (IQR)^a^	488.5 (335–725)	353 (310–401)	376 (310–401)	376 (369–477)	0.52
Serum urea at initiation dialysis (mmol/l), median (IQR)^a^	24.5 (20–30.6)	22 (18.5–22.9)	18.5 (14.4–21.5)	21.1 (17.3–23.7)	0.17
Gestational age at dialysis initiation (weeks), median (IQR)^a^	19 (13–23)	15 (12.5–20.5)^b^	24 (22–24)	19 (13–23)	0.21

*UK* United Kingdom, *SD* standard deviation, *SLE* Systemic Lupus Erythematosus, *CKD* chronic kidney disease, *AVF* Arteriovenous Fistula, *IQR* interquartile range

^a^N = 27; analysis excludes 3 UK cases where AVF was created for dialysis in pregnancy but dialysis was not initiated

^b^Excludes one outlier patient who started dialysis very soon after conception

#### Dialysis initiation

**Fig. 1:**
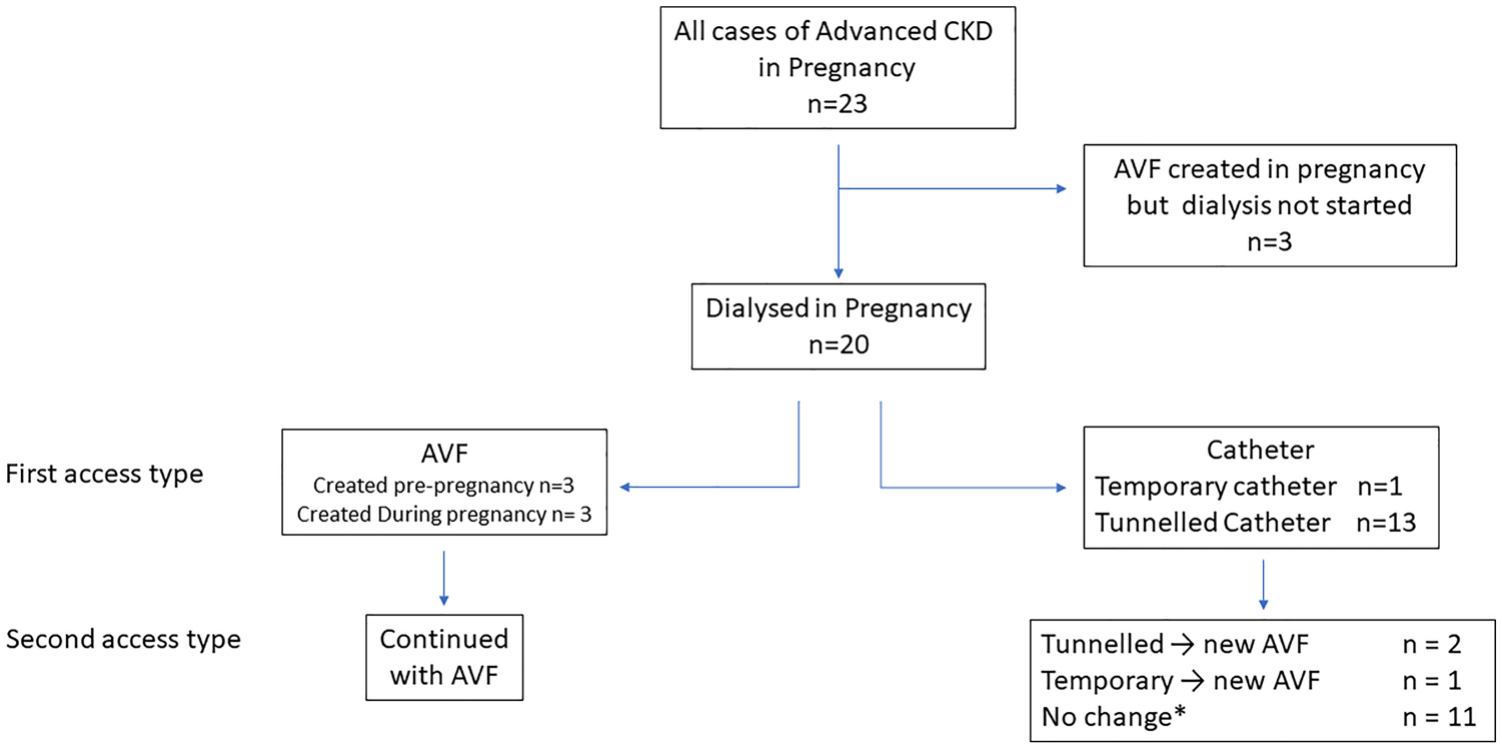
Flowchart of first and subsequent vascular access for dialysis or potential dialysis in pregnancy affected by CKD. *AVF* arteriovenous fistula; *1 case where AVF was created in pregnancy, but dialysis continued via a catheter

New initiation of haemodialysis during pregnancy occurred in 20 women (Fig. 1). In one case the patient received peritoneal dialysis initially and then switched to haemodialysis at 24 weeks’ gestation. The location of dialysis treatment was hospital (n = 13), home including nocturnal (n = 5, all from Canada), and satellite unit (n = 2). The median serum creatinine at dialysis initiation in pregnancy was not significantly different across countries, exhibited a wide range but reflected advanced kidney failure in all cases (Table 1). The overall median gestational age at dialysis start was 19 weeks and was similar among countries. Nineteen of 20 women were still receiving dialysis at 3 months post-partum and only one was dialysis-independent. A further 3 women had an AVF created due to anticipation of haemodialysis during pregnancy but did not commence dialysis due to AVF failure. All 3 women were Caucasian from 3 different centres in the UK and had reflux nephropathy.

#### First access type at haemodialysis initiation: catheters

First and subsequent vascular access types used in women who commenced dialysis are shown in Fig. 1 and comparison of clinical characteristics is summarised in Table 2.

**Table 2:** Maternal parameters according to first vascular access type at dialysis initiation during pregnancy (n = 20 women)

Characteristics	Started with AVF (all cases) N = 6	Started with pre-existing AVF N = 3	Started with new AVF N = 3	Started with catheter N = 14	P value^b^
Maternal age in years, mean (SD)	28.8 (5.9)	30.7 (4.2)	27.0 (7.81)	30.6 (5.4)	0.51
Serum creatinine at initiation dialysis (µmol/l), median (IQR)	426.5 (369–542)	542 (369–823)	376 (335–477)	352.5 (268–625)	0.22
Serum urea at initiation dialysis (mmol/l), median (IQR)	21.4 (18.5–22.9)	22.9 (18.5–28.3)	20.7 (14.3–22)	20.8 (16.1–24.4)	0.93
Gestational age at dialysis start (weeks), median (IQR)	22 (16–23)^a^	14 (12–16)	23 (22–24)	17 (13–23)	0.61

*AVF* arteriovenous fistula, *SD* standard deviation, *IQR* interquartile range

^a^Excludes one outlier patient who started dialysis very soon after conception

^b^Comparing all AVF and catheter groups

Most women (70%) commenced haemodialysis with a catheter, and a vascular catheter was used at any stage during pregnancy in 14 of 20 women (the 6 women who started with AVF never required a catheter). Tunnelled catheters were almost universally used, and none required replacing during the pregnancy. All first catheters were placed in the internal jugular vein. In one patient a temporary catheter was replaced with another temporary catheter due to time in situ > 7 days, following which the patient was dialysed via a newly created AVF. Four women that commenced haemodialysis with a catheter subsequently had an AVF created during the pregnancy (Table 3). Of these 4 cases, 3 women used their AVF during the pregnancy. The remaining patient dialysed via a tunnelled catheter throughout pregnancy and the newly created AVF was only used post-partum.

**Table 3:** Perinatal outcomes according to first maternal vascular access type

	Started with AVF (all cases) N = 6	Started with pre-existing AVF N = 3	Started with new AVF N = 3	Started with Catheter N = 14	AVF with no dialysis N = 3	All N = 23	P value^e^
Gestational age at birth (weeks), median (IQR)	33.5 (31.5–35.5)^a,b^	33^a^^,b^	34 (30–37)	33 (31.1–36.6)^c^	34 (32–37)	33.2 (31.6–36.7)^a,b,c^	0.78
Birth weight (g), median (IQR)	1920 (1535–2490)^a,b^	1760^a^^,b^	2080 (1310–2900)	1780 (1050–2606)^c^	1900 (1690–2790)	1837.5 (1215–2610)^a,b,c,d^	0.53
Live births, n (%)	5 (100.0)^a^	2 (100) ^a^	3 (100.0)	13 (92.9)	3 (100.0)	21 (95.5)^a^	–
Postnatal death, n (%)	0	0	0	1 (7.1)	0	1 (4.5)	
NICU admission for live births, n (%)	3 (60.0)^a^	2 (100.0) ^a^	1 (33.3)	6 (42.9)	1 (33.3)	10 (45.5)^a^	–

*AVF* arteriovenous fistula, *IQR* interquartile range, *NICU* neonatal intensive care unit

^a^Exclusion: one pregnancy termination at 20 weeks in AVF first group

^b^Exclusion: one baby with birthweight of < 500 g in AVF first group

^c^Exclusion: one baby with birthweight of < 500 g in catheter first group; infant died postnatally

^d^Data missing for 2 babies

^e^Started with AVF compared to started with catheter group

#### First access type at haemodialysis initiation: AVF

In total, 6 women commenced dialysis via an AVF; none of these women required a catheter at any stage during pregnancy (Fig. 1). Apart from minor needling problems, all AVFs were used successfully at dialysis initiation with no complications. One patient had sepsis from pyelonephritis. All women remained dialysis-dependent post-partum.

Three women used a pre-existing AVF as their first access for dialysis initiation in pregnancy. The time from creation to conception was 8 months, 18 months and 5.5 years, respectively. In all cases the AVF had been created in anticipation of dialysis but had never been used prior to dialysis initiation in pregnancy. In all cases dialysis commenced within the first 16 weeks of pregnancy, and all women were dialysed in hospital. Blood flow rates were 300 ml/min.

Three women commenced dialysis via a new AVF created during pregnancy, formed at 12, 14 and 15 weeks’ gestation and used at 22, 24 and 30 weeks’ gestation, respectively. One patient initially received peritoneal dialysis and then switched to haemodialysis when the AVF was ready. Blood flow rates for new AVFs were 250–380 ml/min.

Characteristics of women who commenced dialysis via an AVF compared to catheters are shown in Table 2. No statistically significant differences in maternal age, biochemical values or gestational age at dialysis initiation were observed between the AVF and catheter group. We observed that women starting dialysis with a pre-existing AVF commenced at an earlier median gestational age but had higher serum creatinine at dialysis initiation. Women who started with a new AVF created in pregnancy commenced at a later gestational age but with similar biochemical parameters to women commencing with a catheter.

The maximum weekly hours of haemodialysis were calculated for each case and are shown in Table 4 for women with a new AVF created in pregnancy. Weekly dialysis hours varied greatly according to country which also reflected access type. Women in the UK and Australia received 12–24 h/week (irrespective of access type), whereas Canadian women received 24–48 h/week, mostly via home nocturnal dialysis with a catheter.

**Table 4:** Cases of new Arteriovenous Fistula creation during pregnancy

Case	Age (years)	GA at dialysis start (weeks)	Access type at dialysis start	GA at AVF creation (weeks)	GA at first AVF use	Blood flow (ml/min)	Hours of Haemodialysis per week via AVF	Status 3 months post-partum	Complications	Renal status post-partum
1. UK	18	22	AVF	14	22	300	24	Functioning	Pain on initial needling; nil else	Dialysis-dependent
2. UK	32	24	AVF	15	24	380	9	Functioning	Nil	Dialysis-dependent
3. UK	23	14	Catheter	20	Postpartum	Unknown	–	Functioning	Nil	Dialysis-dependent
4. UK	33	Did not start	–	21	–	–	–	Non-functioning	Failed within 1 week	CKD, not on dialysis
5. UK	22	Did not start	–	9	–	1300	–	Non-functioning	Aneurysm and high flow; ligated post-partum	Dialysis-dependent
6. UK	26	Did not start	–	21	–	–	–	Non-functioning	Failed to mature after 6 weeks	CKD, not on dialysis
7. AUS	30	12	Catheter	12	20	300	24	Functioning	Nil	Dialysis-dependent
8. AUS	31	30	AVF	12	30	250	12	Functioning	Needling problems but used successfully	Dialysis-dependent
9. AUS	29	12	Catheter	14	24	162	20–24	Functioning	None	Dialysis-dependent
10. AUS	32	22	Catheter	24	30	130	20	Functioning	Poor blood flows but used successfully	Dialysis-dependent

Cases 3, 7, 9 and 10 had a prior catheter at 14, 12, 12 and 22 weeks’ gestation, respectively. Case 3 presented with AKI and rapidly declining kidney function due to glomerulonephritis and is not included in the cohort of women with advanced CKD

*AVF* arteriovenous fistula, *GA* gestational age (weeks), *ml/min* millilitres per minute

#### Fetal outcomes

There were 22 live births from 23 pregnancies with one medical termination of pregnancy at 20 weeks. The median gestational age and birthweight was similar across all access types including women with AVF created who did not start dialysis (Table 3). Preterm birth, low birth weight and neonatal ICU admission were commonly observed.

### Case series of patients with creation of new AVF during pregnancy

We identified 10 cases where an AVF was constructed during pregnancy (Canada n = 0, UK n = 6, Australia n = 4). Maternal characteristics, timing of AVF creation and use, and AVF outcomes are detailed in Table 4. Nine women had pre-existing advanced CKD at conception and are included in the analysis of women with CKD above. A further case had acute kidney impairment due to rapidly progressive glomerulonephritis developing in pregnancy. All AVFs were constructed under local anaesthesia.

Seven women with newly constructed AVF started dialysis during pregnancy; 4 of these women began with a catheter and all but one went on to use the AVF—this patient delivered before the AVF was mature, and the AVF was used post-partum. Three women had an AVF created during pregnancy at gestational ages 9, 21 and 21 weeks, but did not start dialysis in that pregnancy—in 2 women the AVF failed and in the third the AVF was not used, and eventually became aneurysmal and was ligated post-partum. Salvage of failed AVF was not undertaken as renal function was stable and clinicians determined that dialysis did not need to commence.

### Vascular access complications

Sepsis presumed related to a tunnelled catheter was reported in 1 case at 33 weeks. The patient had presented late in pregnancy (24 weeks’ gestation) and was dialysed via the same catheter until the infective event, which prompted a decision to deliver. There were no reports of local infections, vascular stenosis, poor blood flow or bleeding related to catheter use. Sepsis was reported in one patient with an AVF but was related to pyelonephritis. Needling problems were noted in 3 women with AVF. One patient required single needle dialysis due to bruising, and another AVF had poor blood flow but functioned sufficiently to deliver dialysis during pregnancy. No fistula procedures (fistula revision, angioplasty or stenting) were required or conducted during pregnancy. High output cardiac failure was not reported. Aneurysm formation and cellulitis was reported in one case. This AVF was created at 9 weeks’ gestation and became aneurysmal with high blood flows (1.3 l/min). The patient ultimately did not require dialysis during pregnancy and the AVF was ligated post-partum.

## Discussion

This multi-site, multi-country case series provides new insight into outcomes of vascular access choice for commencing haemodialysis in pregnancy based on experiences of expert obstetric nephrology groups in 3 countries. Catheter use was preferred in the Canadian centre, whereas UK and Australian centres had experience with AVF creation in pregnancy. Catheter use had minimal complications. AVFs were successfully created and used during pregnancy, however primary failure was noted and, in some cases, the AVF was created but not used. The presence of an AVF (pre-existing or new) facilitated catheter removal or avoidance altogether. Maternal parameters and fetal outcomes at dialysis initiation did not significantly differ according to first vascular access type (catheter or AVF).

Commencement of dialysis during pregnancy remains a rare event. In a large UK study of women with non-dialysis dependent Stage 3–5 CKD (including transplanted women), only 3% of women commenced kidney replacement therapy during pregnancy [2]. In Australia from 2000 to 2011, 31% of women receiving chronic dialysis during pregnancy started dialysis in that pregnancy [1]. Efficient, effective and often intensive dialysis therapy is a critical component of care [13, 14] and relies on robust dialysis access. Vascular access practices in non-pregnant cohorts are clinician-, centre- and country-specific [15] and guidelines for care are continuously evolving [16]. Catheter rates for chronic haemodialysis initiation and long-term treatment are 59% and 17% in Australia, and 59% and 35% in the UK [17], respectively. Notably, 60% of prevalent patients in Canada have catheters [18]. US patients are unlikely to start with an AVF, with > 50% long term catheter use [19]. Access choice in the general cohort is linked to clinical outcomes, and should be individualised based on clinical and demographic factors [20] and patient preferences [21, 22].

Not all pregnancies in women requiring dialysis are unplanned. There is now increased “permissiveness” towards dialysis in pregnancy [8], rates of live births in dialysed women are rising [4] and knowledge about best management has evolved [14]. In this new paradigm, women with advanced CKD may undertake shared decision-making with care providers about the best time for them to attempt pregnancy and be supported to achieve pregnancy including planned dialysis start in pregnancy. Vascular access is a critical component of this planning, yet data are minimal. Studies analysing local, registry and hospital data to determine outcomes of dialysis in pregnancy have not reported vascular access [1, 3, 14, 23, 24]. Large cohort studies and a systematic review reporting pregnancy outcome in chronically dialysed women have not reported access-related complications [5, 14, 25], but this does not confirm their absence. We noted very few access-related complications. Temporary catheter use in the non-pregnant dialysis population has been associated with increased infection and mortality [11, 19]. Mehandru et al. reported uncomplicated tunnelled vascular catheter use in 3 pregnancies [10], where women had declined AVF creation for cosmetic and comfort reasons. In our study, 70% of dialysed women had a catheter at some point, and 70% had a catheter as their first access. We observed only one case of catheter-related infection and no tunnelled catheters required replacement during pregnancy. This experience reassures that tunnelled catheters are a safe option for pregnant women.

In our cohort, among all women who commenced dialysis, an AVF (pre-existing or new) avoided a dialysis catheter, and 3 women who commenced with a catheter switched to a newly created AVF during pregnancy. AVF construction during pregnancy is rarely reported but may be an option in suitable women. We identified 10 cases where an AVF was constructed in pregnancy—6 were successfully used in pregnancy. It is important to note that 4 women still required a temporising catheter while awaiting AVF maturation, AVF failed to mature in 2 cases, the AVF was not ready in time in one case, and ultimately 3 women did not start dialysis in the pregnancy. The other AVF-related complications observed in our cohort were minor needling and blood flow issues, but these did not limit AVF use. We have previously reported that first use of AVF in the non-pregnant setting is associated with a high rate of unsuccessful or traumatic cannulation and a third of patients require intervention to improve function [26]. Even with intervention, the overall AVF clinical maturation rate at 6 weeks is approximately 59–75% and there is significant surgeon-level variability in outcome [27, 28]. Concerns regarding aneurysm formation and other complications may dissuade clinicians from creating an AVF in pregnancy. Hormonal changes in pregnancy induce vascular remodelling, and carotid and splenic aneurysms are well-known to occur in pregnancy [29, 30]. There are no data on whether aneurysm formation is accelerated in pregnant women with long-standing pre-existing AVF. One case of aneurysm formation after creation of a left brachial-cephalic fistula at 10 + 3 weeks’ gestation has been reported [31]. We identified 1 new AVF that became aneurysmal in pregnancy and was subsequently ligated without being used.

A further consideration is the total hours of dialysis per week to be delivered during pregnancy, which is dependent on residual renal function and pre-dialysis biochemical targets [13]. Daily or alternate daily dialysis via newly created AVFs may be limited by repeated cannulation trauma and potentially easier with a catheter. Cases in our cohort had widely varying weekly dialysis hours, and women with a new AVF did not receive fewer dialysis hours nor experience problematic cannulation trauma. Overall, centre practice was more influential to access type and hours delivered. The Canadian centre in our study previously demonstrated that extended hours dialysis is associated with better fetal outcomes in women receiving chronic dialysis preconception with minimal residual renal function [14], and most of their patients received extended hours catheter-based nocturnal home dialysis.

Patients and health professionals have prioritised vascular access function as a key outcome in chronic dialysis [22]. Clinician and patient preference, and the urgency of dialysis requirement are the main drivers of vascular access choice. In a subset of women in whom it may be anticipated haemodialysis will start during pregnancy, the option to create an AVF may arise. This includes women with advanced CKD or receiving peritoneal dialysis where a switch to haemodialysis may occur. In our series dialysis commenced at a median gestational age of 19 weeks, giving potential time to consider access options pre-conception or in the early weeks of pregnancy. Pregnancy guidelines recommend education about kidney failure for women with stage 4–5 CKD contemplating pregnancy, but there are no recommendations on vascular access due to limited evidence to underpin choices [13]. AVF formation ahead of pregnancy has been suggested as part of a pre-pregnancy checklist for women already receiving dialysis [32]. There may be concern from clinicians about undertaking an AVF procedure in pregnancy when it is suspected but not guaranteed that dialysis will commence. In women with advanced CKD pre-conception, 7% start dialysis within a year post-partum [2] therefore creation of an AVF may be a reasonable option even if it remains unused in pregnancy. In our cohort most women who had an AVF created in pregnancy remained on dialysis post-partum and the AVF (if successfully created) was eventually used. Furthermore, AVF can be constructed under local anaesthesia, with minimum risk to the patient. These considerations regarding AVF must be balanced against the ease and certainty that a catheter affords. Our study has demonstrated very few catheter-related complications in our cohort, therefore catheter-avoidance need not be pursued aggressively if the clinician and patient preference is for a catheter. Ultimately, these are complex decisions that must be individualised to the context, local practice and setting and patient preference. In women with advanced CKD contemplating pregnancy, shared decision-making about risks, benefits, and potential outcomes for vascular access options in pregnancy should be undertaken.

This study has limitations, mostly related to the retrospective methodology and small cohort size despite gathering cases from 6 centres over a 17-year period. Cases may have not been captured in local datasets or recalled by clinicians if there was no dataset in place. Pregnancy in advanced CKD and AVF formation in pregnancy is a very rare event, and small numbers in this cohort limit detailed sub-analyses and comparisons. Data on pre-pregnancy planning was not available. Cases were derived from tertiary or quaternary centres for obstetric nephrology in highly developed, resource-rich countries and may not reflect care or outcomes in other settings. Differing models of pregnancy healthcare and dialysis care in each country and differing impact of underlying diseases are other important unmeasured confounders.

In conclusion, this study has demonstrated that tunnelled catheters are a safe and reasonable choice for dialysis in pregnancy. In selected women with advanced CKD, AVF can be successfully created preconception or in early pregnancy for use in later pregnancy or post-partum, with low rates of complications. This underscores the importance of early discussion of vascular access options and planning for pregnancy as a critical component of care for women with advanced CKD.

## Data Availability

The data underlying this article cannot be shared publicly due to risks to privacy of individuals whose cases are presented.
